# Application of laser capture microdissection and PCR sequencing in the diagnosis of *Coccidioides* spp. infection: A case report and literature review in China

**DOI:** 10.1080/22221751.2021.1889931

**Published:** 2021-02-27

**Authors:** Xinyu Yang, Yinggai Song, TianYu Liang, Qiqi Wang, Ruoyu Li, Wei Liu

**Affiliations:** Department of Dermatology and Venereology, National Clinical Research Center for Skin and Immune Diseases, Research Center for Medical Mycology, Beijing Key Laboratory of Molecular Diagnosis on Dermatoses, Peking University First Hospital, Beijing, People’s Republic of China

**Keywords:** Coccidioidomycosis, *Coccidioides* spp, laser capture microdissection, molecular diagnosis

## Abstract

Coccidioidomycosis is endemic to California, Arizona, and Mexico. In recent years, the reported cases of coccidioidomycosis have increased in nonendemic regions. Here, we reported a case of imported pulmonary coccidioidomycosis in a Chinese patient. A 63-year-old man presented with dry cough and fatigue for 6 months, and a computed tomography scan revealed a solitary nodule in the right lower lung and small nodules in both lungs. The diagnosis of coccidioidomycosis was initially confirmed by histopathologic examination. The pathogen *Coccidioides* spp. was identified by laser capture microdissection (LCM) combined with subsequent molecular techniques based on the positive histopathologic features. Additionally, we reviewed 47 reported cases of coccidioidomycosis in China. The number of reported cases is increasing, and the incidence of disseminated infection has exhibited a trend of shifting towards healthy young adults in China. Since clinical presentations and imaging findings lack specificity, a majority of domestic cases of coccidioidomycosis were initially misdiagnosed as tumours or tuberculosis. Moreover, the diagnosis of endemic mycoses may be challenging because of their rarity and the limited availability of diagnostic tests. The diagnosis was mainly confirmed by histopathological examination. The species involved were identified based on positive cultures in only 4 cases. To our knowledge, this is the first study to use LCM and molecular techniques to identify *Coccidioides* spp. in the histopathologically positive but uncultivable specimen. Comparing with previous reported studies, LCM combined with nucleic acid amplification techniques improve the ability of species identification for the timely diagnosis of coccidioidomycosis.

## Introduction

Coccidioidomycosis is a fungal disease caused by *Coccidioides immitis* and *Coccidioides posadasii.* Coccidioidomycosis is endemic to the Americas, including South America. Most cases are reported in California and Arizona, but it is found in many arid regions throughout North and South America where it is underreported [1]. With the continuous development of international communication and tourism, reported imported cases have been increasing in nonendemic areas [2]. Coccidioidomycosis can clinically manifest as an asymptomatic infection, primary pulmonary infection or even a disseminated infection [1]. Since the disease is rare and its clinical signs are nonspecific, it is easily misdiagnosed as a tumour or other disease in nonendemic areas.

The diagnosis of coccidioidomycosis is mainly based on exposure history, clinical manifestations and laboratory tests [3]. Coccidioidomycosis should be considered in the differential diagnosis of patients who have respiratory, skin, neurological and other symptoms and have recently travelled to endemic areas. Laboratory tests for the disease include histopathological examination, the isolation and culture of pathogens, serological examination, and the use of molecular methods for species identification [1,3]. Fungal culture and histopathology analyses are the gold standards for the diagnosis of coccidioidomycosis. The histopathology of coccidioidomycosis is characterized by spherules 20–200 μm in diameter containing endospores observed via microscopy [3]. Periodic acid-Schiff (PAS) and Gomori methenamine silver (GMS) staining is very sensitive for detecting a small number of spherules. Fungal culture usually requires 3 weeks and should be carried out in a biosafety level 3 laboratory (BSL-3), which may lead to delayed diagnosis and potential laboratory transmission [3]. Additionally, in nonendemic areas, serologic testing is not routinely available [3].

If clinical specimens cannot be cultured, the application of molecular techniques to detect *Coccidioides* spp. directly from formalin-fixed, paraffin-embedded (FFPE) tissue is useful for the molecular diagnosis of coccidioidomycosis [4]. For example, a specific target region of the internal transcribed spacer (ITS) or the proline-rich antigen (PRA) coding region of the fungal genome may be amplified by PCR for species identification. Furthermore, Real-time PCR is a rapid and sensitive method for analyzing single-nucleotide polymorphisms (SNPs) from polymorphic gene fragments to identify possible species [5].

In recent years, laser capture microdissection (LCM)-based analytic techniques have been applied for the microbiological diagnosis of infectious diseases [6, 7]. LCM is a novel and powerful technique for selectively harvesting cells of interest directly from histological sections under direct microscopy [8]. It is widely used for genomic analysis, gene expression profiling and the evaluation of host-microorganism interactions [9].

In this study, we reported a case of imported coccidioidomycosis confirmed by histopathology. To further determine the pathogen, suspected pathogenic fungi from FFPE lung tissue sections subjected to PAS staining were collected by LCM. Genomic DNA was extracted, and downstream PCR and Sanger sequencing were then performed to identify the pathogen. Since coccidioidomycosis is rare in China, we summarized all reported 47 cases so as to better understand the etiology and clinical features of this disease.

## Case

**Figure 1.**
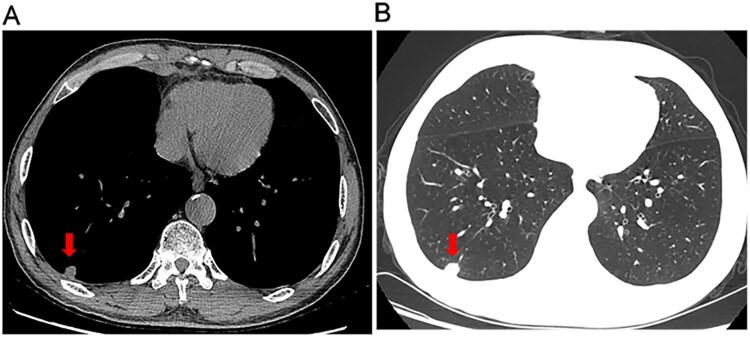
Chest computed tomography (CT) scans. CT images of the lung window (A) and soft tissue window (B) revealed a 1.4 × 1.3 × 1.1 cm solitary solid nodule in the left lower lobe (arrowhead), mild ground-glass opacity and multiple small nodules in both lungs.

**Figure 2.**
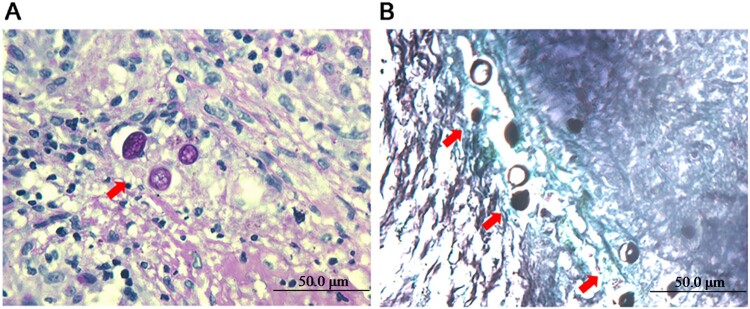
Histopathological examination of biopsy tissue. PAS staining (A) and GMS staining (B) showing multiple thick-walled spherules and endospores (arrowhead) in the nodules.

A 63-year-old man presented to our hospital with a 6-month history of dry cough and fatigue. Six months earlier, the patient had presented with dry cough and fatigue without an obvious cause. He did not exhibit fever, headache, or night sweats. A chest computed tomography (CT) scan at a local hospital revealed a solitary solid pulmonary nodule in the right lower lobe. The patient was suspected of having bacterial infection and was treated with intravenous cephalosporin for 10 days but showed no improvement. He was previously healthy except for a 20-year history of asthma. One year prior to his appearance at the hospital, he had worked as a miner for 6 months in Mexico. Laboratory tests showed decreased levels of total protein (60.6 g/L), albumin (33.6 g/L), and alkaline phosphatase (33 IU/L), and the results of other routine laboratory examinations were normal. Repeated chest CT revealed a 1.4 × 1.3 × 1.1 cm solitary solid nodule with a smooth margin in the right lower lobe and multiple small 0.1- to 0.2-cm nodules with mild ground-glass opacity in the bilateral lungs (Figure 1A, B). Histological examination of biopsy tissue from a thoracoscopic right lower lobectomy revealed multiple granulomas with caseous necrosis, in which scattered thick-walled spherules containing endospores were observed following PAS and GMS staining (Figure 2A, B). Serum galactomannan (GM) and 1,3-beta-D-glucan (G) assays and *Mycobacterium tuberculosis* culture results were negative. Therefore, a diagnosis of pulmonary coccidioidomycosis was established. The patient was treated with oral fluconazole (400 mg per day) for 6 months. The symptoms were significantly relieved, and the pulmonary nodules observed in CT images became progressively smaller and eventually disappeared during treatment. The patient is still undergoing follow-up.

## Materials and methods

### Laser capture microdissection of FFPE tissue sections

To further identify the pathogen at the species level, FFPE tissue was cut into 6 μm-thick sections, which were then affixed to microscope slides, precoated with polyethylene naphthalate, for PAS staining. Laser microdissection and laser pressure catapulting were performed to capture PAS-positive fungal components such as hyphae and spores in the tissue sections under light microscopy using a Lecia LMD6000 system (Leica, Wetzlar, Germany) in a laminar flow biosafety cabinet. Pure cells or tissues on the slides were dissected with a 337 nm pulsed ultraviolet laser and collected in a sample tube. As a negative control, an adjacent nonfungal tissue sample of similar size was excised from the same specimen and processed in parallel [6].

### Extraction of fungal genomic DNA from LCM-isolated FFPE tissue and PCR amplification

Genomic DNA was extracted from LCM-isolated FFPE tissue using the QIAamp DNA Mini Kit (Qiagen, Hilden, Germany) following the manufacturer’s instructions. The universal fungal ITS primers ITS1 (5’-TCCGTAGGTGAACCTGCGG-3’) and ITS4 (5’-TCCTCCGCTTATTGATATGC-3’) were used as outer primers for the first round of PCR [10]. PCR was carried out in a 25 μl volume containing 20 ng of genomic DNA, each of the primers at 0.08 μM, and 12.5 μl of 2× Taq PCR MasterMix (Tiangen Biotech Ltd, Beijing, China). The PCR conditions were as follows: initial denaturation at 94°C for 5 min, followed by 35 cycles of denaturation at 94°C for 30 s, annealing at 55°C for 30 s, and extension at 72°C for 1 min, with a final extension step at 72°C for 5 min. Then, the PCR products were used as templates for nested PCR with the *Coccidioides*-specific primers ITSC1 (5’-CATCATAGCAAAAATCAAAC-3’) and ITSC2 (5’-AGGCCCGTCCACACAAG-3’) [11]. The cycling conditions were as follows: initial denaturation at 94°C for 2 min; 35 cycles of 94°C for 1 min, 53°C for 1 min, and 72°C for 1 min; followed by a final extension at 72°C for 7 min. *A. fumigatus* genomic DNA and sterile water were used as templates to validate the specificity of the nested PCR assay.

### Cloning and sequencing of amplified products

The PCR products from each round of PCR were ligated with T-Vector pMD™19 (Takara, Otsu, Japan) using T4 DNA ligase (Takara, Otsu, Japan). The ligated products were then transformed into *Escherichia coli* DH5α competent cells (Yeasen, Shanghai, China). The transformed cells were plated on LB agar plates containing 100 μg/ml ampicillin and incubated at 37°C for 16–18 h. Single colonies were then randomly picked. Sixty positive clones from the first-round PCR products and 12 positive clones from the second-round PCR products were screened by colony PCR. Then, the 72 colonies were individually inoculated into LB liquid broth with 100 μg/ml ampicillin and grown at 37°C with shaking at 220 rpm for 12 h. The plasmids were subsequently extracted using the TIANprep Mini Plasmid Kit (Tiangen Biotech Ltd, Beijing, China) and sent to the BGI Company (Beijing, China) for sequencing using the M13F (5’-CAGGAAACAGCTATGAC-3’) and M13R (5’-GTTTTCCCAGTCACGA-3’) primers [12]. The sequences of the inserted fragment were aligned against the CBS database (https://wi.knaw.nl/page/Pairwise_alignment) and GenBank database (https://blast.ncbi.nlm.nih.gov/Blast.cgi?PROGRAM=blastn&PAGE_TYPE=BlastSearch&LINK_LOC=blasthome) for species identification.

### Real-time PCR assay using Coccidioides-specific TaqMan probes

Real-time PCR is a highly sensitive and specific method for quickly detecting *Coccidioides* directly from clinical specimens [13]. Castano-Olivares et al. [14] described a method in which a probe labelled with the VIC^TM^ fluorophore (5’-VIC-CCCAATTGAGATCCCA-3’) was hybridized to nucleotides 404–467 of the *C. immitis PRA* gene and a probe labelled with the FAM^TM^ fluorophore (5’-6FAM-CCCAATTGACATCCCA-3’) was hybridized to nucleotides 415–478 of the *C. posadasii PRA* gene. Genomic DNA extracted from LCM-isolated tissue was used as the template for Real-time PCR with the primers PRAF1 (5’-GTCGTTGACCAGTGCTCCAA-3’) and PRAR1 (5’-CGGCGGTGGTGTCAACT-3’) and the abovementioned specific probes. An Applied Biosystems ViiA7 Real-time PCR system was used. Each reaction mixture volume (20 μl) contained 1× ABI TaqMan PCR Master Mix (Applied Biosystems, Rochester, NY), each primer at 900 μM, 2 μl of template, and each fluorescein-labelled probe at 125 μM. The cycling conditions were as follows: an initial denaturation step at 94°C for 10 min, followed by 50 cycles of denaturation at 92°C for 15 s and annealing/elongation at 60°C for 60 s. All samples were run in triplicate.

### Literature review of coccidioidomycosis in China

We searched the literature on coccidioidomycosis in Chinese patients from PubMed (http://www.ncbi.nlm.nih.gov), the China Knowledge Resource Integrated (CNKI) database (http://www.cnki.net), and the Wanfang database (http://www.wanfangdata.com.cn/index.html) published between 1958 and 2020. The search terms used were “coccidioidomycosis” and “China”. The epidemiological features, underlying diseases, clinical manifestations, diagnostic methods, treatment and prognosis of all patients were summarized and analyzed.

## Results

### Fungi were isolated by LCM from FFPE tissue sections

**Figure 3.**
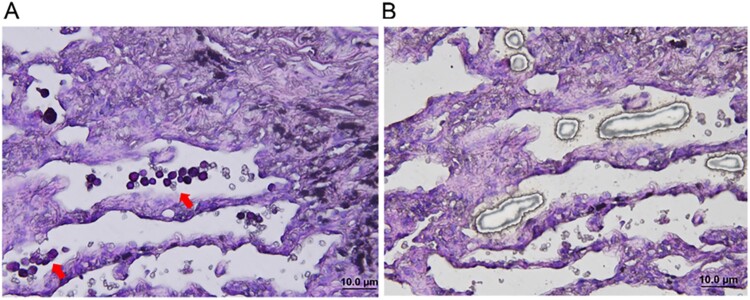
Isolation of fungi from formalin-fixed and paraffin-embedded (FFPE) tissue using LCM. (A) Small, round purpled-red fungal spores (arrowhead) scattered within alveoli were visible in the PAS-stained section before LCM. (B) Fungal spores were microdissected and collected after LCM.

Round purple-red fungal spores scattered within alveoli were visible in the PAS-stained FFPE tissue sections under the inverted microscope of an LCM instrument (Figure 3A). Fungal spores were further microdissected under UV light and collected in a sterile environment (Figure 3B).

### Coccidioides and other filamentous fungi were detected by PCR in LCM-isolated FFPE tissue

**Figure 4.**
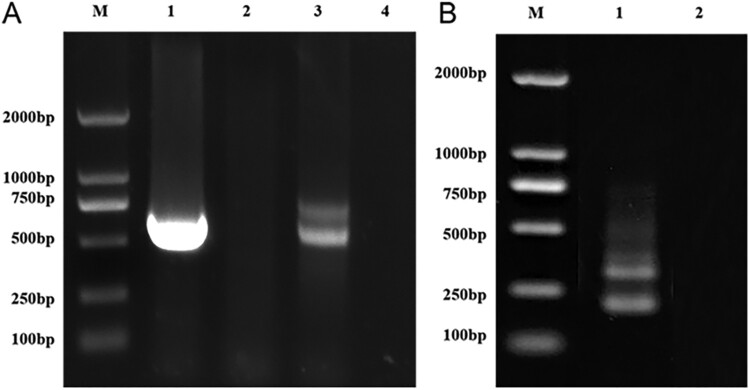
Electrophoresis of the nested-PCR amplification products in 2% agarose gels. (A) DNA products amplified in first-round PCR using the universal primers ITS1 and ITS4. Lane M, 2 kb DNA ladder. Lane 1, product amplified from *Aspergillus fumigatus* DNA as positive control. Lane 2, negative control, no amplification product by using DNA extracted from laser microdissected adjacent areas without fungal spores in the identical field of the same tissue section. Lane 3, products amplified from fungal DNA extracted from LCM-isolated FFPE tissue. Lane 4, no-template control. (B) Nested PCR products obtained by using the first-round PCR products as templates and ITSC1 and ITSC2 as the *Coccidioides*-specific primers. Lane M, 2 kb DNA ladder. Lane 1, nested PCR products obtained by using the first-round PCR products as templates. Lane 2, no product obtained by using first-round PCR products, amplified from *A. fumigatus* DNA, as templates.

Using the universal fungal primers ITS1 and ITS4, the first-round PCR yielded two bands between approximately 500 and 750 bp (Figure 4A). The DNA in the two bands was purified and cloned. Sixty positive clones were selected for plasmid DNA extraction and sequencing. A variety of fungal species were identified, including *Alternaria* spp., *Aspergillus* spp., *Cladosporium* spp., and *Fusarium* spp.

At the same time, the products from the first-round PCR were used as the templates and the ITSC1 and ITSC2 were used as *Coccidioides*-specific primers for nested PCR amplification, yielding two bands of approximately 250 bp (Figure 4B). Each of the PCR products was purified and cloned. Twelve positive colonies were sequenced. A 237-bp sequence of the inserted fragment obtained from positive colonies was deposited in the NCBI database under GenBank accession number MT899215, which showed 97.92% similarity with *C. immitis* sequences in both CBS and GenBank databases.

### Coccodioides spp. was detected by Real-time PCR in LCM-isolated FFPE tissue

**Figure 5.**
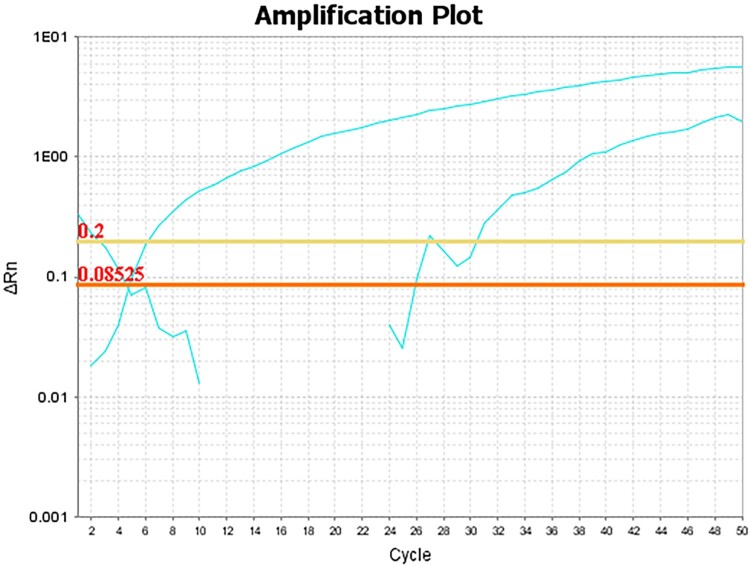
Amplification curves of TaqMan-based Real-time PCR assay using *Coccidioides*-specific TaqMan probes and fungal DNA extracted from LCM-isolated FFPE tissue as template.

The Real-time PCR results showed the two amplification curves presented in Figure 5. The cycle threshold (Ct) value of the first amplification curve labelled with the VIC fluorophore was 6, possibly corresponding to *C. immitis*. The Ct value of the other amplification curve labelled with the FAM fluorophore was 30, possibly corresponding to *C. posadasii*. And the amount of *C. immitis* may be greater than that of *C. posadasii* since the Ct value of *C. immitis* was significantly lower than that of *C. posadasii* [15]. These results suggest that both *C. immitis* and *C. posadasii* may present in LCM-isolated FFPE tissue.

### Literature review of coccidioidomycosis in China

Following literature review, 38 articles reporting 47 coccidioidomycosis patients were included in this study (Table 1). The new patient reported herein was also included, leading to a total of 48 patients [16–52]. Twenty patients (42.6%) had a definitive history of travel or residence in endemic areas, 22 patients (46.8%) reported no exposure history, and 5 patients had unknown exposure history.

**Table 1.** Coccidioidomycosis cases reported in China from 1958 to 2020.

	Year	References	Age/Sex	Provinces	Job	Underlying disease	Site of infection	Travel history	Method of identification	Treatment	Outcome
1	1958	[16]	35/male	Guangdong	Pilots	None	Skin	California	Biopsy/Culture	Surgery	Cured
2	1963	[17]	43/male	Shandong	Merchant	NM	Disseminated(Lung/Skin)	None	Culture	Antibiotics	Died
3	1985	[18]	31/male	Jiangxi	Autoworker	None	Lung	None	Culture	CTZ	Remission
4	1985	[18]	32/female	Jiangxi	Autoworker	None	Lung	None	Culture	CTZ	Remission
5	1988	[19]	26/female	Shanghai	Weaver	None	Disseminated(Lung/CNS/ Vertebrae/Spleen/Liver)	None	Biopsy	KTZ	Cured
6	1991	[20]	28/male	Taiwan	NM	NM	Disseminated(Lung/Skin/Lymph node)	Arizona	Biopsy	KTZ	Cured
7	1995	[21]	45/female	Guangdong	NM	NM	cornea	None	Biopsy	AMB	Cured
8	1998	[22]	32/male	Hongkong	NM	None	Disseminated(Lung/Skin/Lymph node/Bone)	California	Biopsy/Culture	AMB/FLU/ITC	Remission
9	1998	[23]	52/male	Guangdong	NM	HIV	Skin	None	Biopsy	NM	NM
10	1999	[24]	37/male	Jiangsu	prisoner	Drug abuse	Disseminated(Lung/CNS/Spleen)	None	Biopsy	NM	Died
11	2002	[25]	44/female	Hubei	Worker	NM	Lung	None	Biopsy	Surgery	NM
12	2003	[26]	49/female	Zhejiang	Farmer	NM	Bone	None	Biopsy	NM	NM
13	2008	[27]	5/male	Hunan	NM	None	Disseminated(Lung/Liver/CNS/Spleen/Skin)	NM	Biopsy	None	Died
14	2008	[28]	38/male	Shandong	NM	None	Skin	USA	Biopsy	Surgery	NM
15	2008	[29]	14/male	Hubei	Student	None	Lung	NM	Biopsy	AMB	Remission
16	2009	[30]	75/male	Zhejiang	NM	None	Lung	Arizona	Biopsy	Surgery	NM
17	2009	[31]	33/male	Jiangsu	Worker	NM	Disseminated(CNS/Lymph nodes)	NM	Biopsy/Antibody	FLU	Cured
18	2009	[32]	50/male	Hongkong	NM	None	Lymph node	California	Biopsy	FLU	Cured
19	2009	[32]	42/male	Hongkong	Jewellery worker	Chronic active hepatitis B/Nasopharyngeal cancer	Disseminated(Lung/Ribs/Vertebrae)	California	Culture	FLU/AMB	Died
20–27	2010	[33]	Mean 43 Male/female	Fujian	NM	None(7) HIV(1)	Lung	None	Biopsy	AMB/FLU/ITR	Remission(7) NM(1)
28	2010	[34]	74/male	Jiangsu	NM	Hypertension	Lung	None	Biopsy	FLU + VRC	Cured
29	2010	[35]	14/male	Hubei	NM	None	Lung	NM	Biopsy	FLU/AMB	Cured
30	2010	[36]	58/female	NM	NM	NM	Lung	NM	Biopsy	FLU	Remission
31	2010	[37]	81/male	Zhejiang	Farmer	Chronic bronchitis	Lung	None	Biopsy	FLU	Remission
32	2011	[38]	71/male	Zhejiang	NM	Hypertension/Coronary heart disease.	Lung	Arizona	Biopsy	Surgery	Cured
33	2011	[39]	43/male	Hongkong	Driver	NM	Skin	None	Biopsy/Antibody	FLU	Cured
34	2016	[40]	24/female	Beijing	Student	None	Disseminated(Lung/Lymph node)	USA	Biopsy	Antifungal	Remission
35	2016	[41]	51/female	Guangdong	NM	NM	Stomach	None	Biopsy	FLU	Remission
36	2016	[42]	49/female	Shanxi	NM	Nephrotic Syndrome	Lung	None	Biopsy	Surgery	Cured
37	2016	[43]	61/male	Zhejiang	NM	Hypertension	Lung	Arizona	Biopsy	FLU	Remission
38	2017	[44]	24/female	Beijing	NM	None	Disseminated(Lung/Lymph node/Bone)	USA	Biopsy	ITC/AMB/ DEX	Remission
39	2017	[45]	27/male	Jiangsu	NM	NM	Disseminated(Lung/Lymph node/Bone/CNS)	Arizona	Biopsy/Culture	ITZ/FLU	Remission
40	2017	[46]	28/male	Fujian	pilot	None	Disseminated(Lung/Lymph node/Bone/CNS/Vertebrae)	USA	Biopsy/Culture	ITZ/FLU	Remission
41	2018	[2]	29/male	Jiangsu	Student	None	Lung	Arizona	Culture/PCR	VOR	Remission
42	2018	[47]	20/female	Beijing	Student	None	Disseminated(Lymph node)	California	Biopsy	AMB/ITR	Cured
43	2018	[48]	55/male	Guangdong	NM	NM	Lung	California	Biopsy	FLU	Cured
44	2018	[59]	22/female	Jiangsu	Student	None	Lung	USA	Biopsy	FLU	Cured
45	2019	[50]	61/male	Shanghai	NM	NM	Disseminated(Lung/Lymph node/Skin)	USA	Culture/MALDI-TOF MS	FLU	Remission
46	2019	[51]	55/male	Zhejiang	University Professor	None	Disseminated(Lung/Lymph node)	Arizona	Culture/MALDI-TOF MS/NGS	VOR	Cured
47	2019	[52]	24/male	Guangxi	Student	None	Disseminated(Skin/Bone/Vertebrae/Lung/Lymph node)	Los Angeles	Culture/Biopsy/NGS	FLU/VOR/ITC	Remission
48	2020	Here	63/male	Hebei	Miner	Asthma	Lung	Mexico	Biopsy/LCM/PCR/Real-time PCR	Surgery/FLU	Remission

FLU, fluconazole; AMB, amphotericin B; VOR, voriconazole; ITC, itraconazole; CTZ, clotrimazole; KTZ, ketoconazole; DEX, dexamethasone; CNS, central nervous system; MALDI-TOF MS: matrix-assisted laser desorption/ionation time of flight mass spectrometry; NGS: next-generation sequencing; LCM: laser capture microdissection; NM, not mentioned.

Among the 47 patients, the most common infection site was lung and 37 patients (78.73%) had respiratory symptoms such as dry cough or white, yellow, bloody sputum. Thirty-one patients (66.0%) showed single system involvement, including 23 cases of lung infection, 4 cases of skin infection, and 1 case each of lymph node, cornea, stomach, and bone infection. In addition, 16 patients (34.0%) presented a disseminated infection involving the lungs, lymph nodes, central nervous system and other organs, 8 of which (50.0%) had been reported during the past 5 years (2016–2020). Disseminated infections were severe and mainly occurred in young individuals (87.5%). Additionally, 8 patients (17.0%), including 6 patients with single system involvement and 2 patients with disseminated infection, exhibited underlying diseases such as acquired immune deficiency syndrome (AIDS), drug abuse, chronic hepatitis and other diseases. However, 26 patients (55.3%) had no underlying diseases.

Chest imaging of pulmonary infection showed nodules, cavities, masses, hilar and peritracheal lymphadenopathy, diffuse ground-glass opacity and consolidation in the lung. X-ray or MRI visualization of bone infection mainly revealed severe osteolytic bone lesions and bone destruction. All of these radiologic findings of coccidioidomycosis were nonspecific.

The laboratory diagnoses of coccidioidomycosis cases in China were mainly based on histopathology, which was positive in 40 cases (85.1%) (Table 2). The pathogenic species were identified on the basis of positive culture in only 4 cases: two of the patients infected by *C. posadasii* were diagnosed by PCR or a combined matrix-assisted laser desorption/ionation time of flight mass spectrometry (MALDI-TOF MS) and next-generation sequencing (NGS), the third patient infected by *C. immitis* was identified by histopathology and NGS, and the last one infected by mixed infection of *C. immitis* and *C. posadasii* was identified using MALDI-TOF MS. Based on histopathologic features, *Coccidioides* spp. was identified by LCM combined with nested PCR and Real-time PCR in our patient.

**Table 2.** Summary of diagnostic methods of 48 cases of coccidioidomycosis.

Diagnostic methods	Number	Fungal species
Histopathology alone	33	*Coccidioides* spp.
Fungal culture alone	4	*Coccidioides* spp.
Histopathology + Fungal culture	4	*Coccidioides* spp.
Histopathology + Serology	2	*Coccidioides* spp.
Fungal culture + PCR	1	*C. posadasii*
Fungal culture + MALDI-TOF MS + NGS	1	*C. posadasii*
Fungal culture + MALDI-TOF MS	1	*C. immitis *+ *C. posadasii*
Histopathology + Fungal culture + NGS	1	*C. immitis*
Histopathology + LCM + PCR sequencing		
(Here)	1	*Coccidioides* spp.

MALDI-TOF MS: matrix-assisted laser desorption/ionation time of flight mass spectrometry; NGS: next-generation sequencing; LCM: laser capture microdissection.

Among the 47 patients, patients with single system involvement were usually treated with fluconazole alone and those with disseminated infections were usually treated with fluconazole in combination with amphotericin B. And the remission or cure rate was 94.5%. Six patients underwent surgery, 3 of whom were cured, while the results for other 3 were not reported. Another three patients with disseminated infections did not receive antifungal treatment because of not considering the diagnosis of coccidioidomycosis, and died.

## Discussion

In this study, the patient developed nonspecific manifestations such as dry cough, fatigue, and pulmonary nodules, which were in consistent with domestic cases of pulmonary coccidioidomycosis. The diagnosis of coccidioidomycosis was confirmed by the presence of spherules containing endospores using histopathologic examination, and *Coccidioides* spp. was identified in limited FFPE tissue using a combination of LCM, nested PCR and Real-time PCR. Luna-isaac *et al.* [53] studied the relationship between *Coccidioides* species and their clinical manifestations as well as geographical distribution in Mexico, and he reported that *C. immitis* and *C. posadasii* were mainly distributed in northeastern and northwestern Mexico, respectively [53]. These two organisms were genetically distinct but unable to be distinguished on the basis of morphological and clinical features, immune response, or epidemic regions [1, 53]. Therefore, we speculated that this patient reported herein may inhale two species of *Coccidioides* spores during long-term contact with soil.

Additionally, round PAS-positive fungal microconidia, which may be species of *Coccidioides* spp., *Aspergillus* spp., *Penicillium* spp., or other fungi in the stained tissue, were shown in Figure 3. Hence, these microconidia cannot be identified as *Aspergillus* spp. alone, since it is difficult to identify species of microconidia based on the round PAS positive appearance observed on the tissue section. The histopathological features of *Coccidioides* in the biopsy specimens are spherules filled with 2- to 4-μm-diameter endospores. The mycelial form is very uncommon [3]. Hence, we believe that the presence of fungal conidia without fruiting bodies or hyphae in FFPE tissue is mostly the endospores released from those broken spherules. For *A. fumigatus*, conidia can bind to lung type II alveolar epithelial cells and invade the cells by inducing their own internalization, and gliotoxin might be responsible for modulating *A. fumigatus* internalization into epithelial cells through phospholipase D (PLD) activation and actin cytoskeleton rearrangement. Consequently, *A. fumigatus* conidia survive and disseminate within these normally non-phagocytic host cells upon evasion of host defense by phagocytes, and then colonize the respiratory tissue and initiate vascular diffusion [54–56]. Previous studies showed that 70% of severe asthma in adult patients was caused by fungal sensitization [57]. The predominant etiologic agents, including the species of *Aspergillus*, *Alternaria*, *Cladosporium*, *Penicillium*, and *Fusarium* were also identified in FFPE tissue from this patient, which could cause asthma in this patient for up to 20 years since these fungi were speculated colonizing the respiratory tract and triggering IgE-mediated allergic reactions [57, 58].

A total of 47 reported cases of coccidioidomycosis in China were collected. Ten cases were reported from 1958 to 2000, 9 cases were reported from 2000 to 2009, and 28 cases were reported from 2010 to 2019, showing the increased incidence of coccidioidomycosis in China. In endemic areas, individuals with AIDS, diabetes mellitus, and those who have received organ transplantation are believed to be high-risk groups [1]. Farmers and construction workers who are in frequent contact with soil also exhibit a high incidence rate [1]. However, 26 patients (55.3%) had no underlying diseases among 47 Chinese patients. Furthermore, disseminated infection mainly occurred in previously healthy young individuals in China, unlike those who are more likely occurrence in immunosuppressed patients reported in endemic areas, and the high incidence rate was 53.4% during the past 5 years [1, 3]. Therefore, more of epidemiological studies on the risk factors and underlying diseases for coccidioidomycosis need to be further investigated in China. Additionally, 22 (46.8%) patients have no history of exposure to endemic areas or contact with patients or individuals returned from the endemic regions, suggesting that native *Coccidioides* may exist in China.

Among the 47 patients, the most common clinical features were cough and fever, which were nonspecific and consistent with those reported in endemic areas. A majority of domestic cases were initially misdiagnosed as tumours or tuberculosis. Therefore, more attention should be paid to the precise diagnosis of coccidioidomycosis. The pathogenic species were only identified in 4 patients using a combination of culture and subsequent molecular diagnostic techniques such as PCR, NGS, or MALDI-TOF MS. In our patient, *Coccidioides* spp. was identified in FFPE tissue by LCM combined with nested PCR and Real-time PCR based on histopathology. Currently, the LCM technique can isolate precise cell populations or microorganisms from complex clinical FFPE samples, avoiding the cross-contamination of surrounding heterogeneous tissues in the specimen [6]. This method and subsequent molecular techniques have been used to isolate pure tumour tissues and detect the expression of target genes, which was initially applied for analyzing cancers [6]. In a previous study, *Trichoderma* DNA in FFPE specimens was detected using the combined LCM and PCR technique, resulting in the clinical diagnosis of rare *Trichoderma* infections [8]. Whereas the formalin fixation and paraffin embedding process can cause damage to nucleic acids such as DNA fragmentation, degradation, and chemical modification of the bases [59]. Therefore, it is important to improve DNA extraction yields. Nested PCR and Real-time PCR techniques have been proven to increase the sensitivity and specificity of detecting microorganism nucleic acids in FFPE tissues. For example, Schofield *et al*. [59] reported that the sensitivity of nested PCR was 1,000 times higher than that of conventional PCR in the detection of *Candida albicans*, and Binnicker *et al.* [13] reported that the specificity of Real-time PCR was 100% in the detection of *Coccidioides* spp. in FFPE tissues. Therefore, we used a combination of LCM, PCR and Real-time PCR to detect and identify *Coccidioides* spp. in the limited number of FFPE samples, which improved the specificity and sensitivity of detection comparing with the conventional method of DNA extraction from FFPE tissues.

Since *C. posadasii* had not been split from *C. immitis* until 2002 [60], the *C. immitis*-specific primers described in 2000 [11] were in fact able to amplify both *C. immitis* and *C. posadasii* DNA. So, the result of the nested PCR that being performed with the primers according to this literature [11] just suggested the presence of *Coccidioides* spp. in FFPE tissue. We hence speculated that the pathogen may be *C. immitis*, or *C. posadasii*, or the mixture of these two species. In addition, a Real-time PCR assay using specific TaqMan probes labelled with VIC and FAM fluorescence was reported to identify and distinguish the clinical isolates of *C. immitis* and *C. posadasii* by Castañón-Olivares et al. in 2007 [14]. However, this method has not been used to detect *Coccidioides* spp. from FFPE tissue, yet. And because of lacking the strains stock of *Coccidioides* spp. in our laboratory, we were unable to evaluate the specificity and sensitivity of this Real-time PCR method in detecting *C. immitis* and *C. posadasii* in this study. Therefore, we detected *Coccidioides* spp. directly by performing the Real-time PCR assay as described previously [14] with the very limited amount of DNA that being extracted from the FFPE tissue. And the result showed there were two amplification curves, indicating the two species of *C. immitis* and *C. posadasii* may be existed in FFPE tissue. Taken together, the diagnosis of coccidioidomycosis for this patient has been confirmed by histopathological examination combined with the detection of *Coccidioides* spp. in FFPE tissue with further LCM and PCR sequencing strategies.

In summary, we identified a case of imported coccidioidomycosis and analyzed all 47 cases of coccidioidomycosis reported in China since 1958. The incidence rates of coccidioidomycosis in China have been increasing in recent years. In 47 reported cases, *Coccidioides* spp. was unable to be identified in the clinical specimens that unable to be cultured or culture negative. This patient described here is the first study to identify *Coccidioides* spp. using LCM in combination with PCR sequencing strategies based on histopathological examination, which have been proven being with good safety and sensitivity in clinical application. We believe that this method will be used for the identification of *Coccidioides* spp. directly in FFPE tissues, allowing for the timely diagnosis of coccidioidomycosis and the appropriate initiation of antifungal therapy.
